# Behavioural Ecology and Group Cohesion of Juvenile Western Lowland Gorillas (*Gorilla g*. *gorilla*) during Rehabilitation in the Batéké Plateaux National Park, Gabon

**DOI:** 10.1371/journal.pone.0119609

**Published:** 2015-03-26

**Authors:** Guillaume Le Flohic, Peggy Motsch, Hélène DeNys, Simon Childs, Amos Courage, Tony King

**Affiliations:** 1 The Aspinall Foundation, Projet Protection des Gorilles, Franceville, Gabon; 2 African Parks Network, Parc National d'Odzala-Kokoua, Unité de Gestion, Brazzaville, République du Congo; 3 Centre International de Recherches Médicales de Franceville, Franceville, Gabon; 4 The Aspinall Foundation, Port Lympne Wild Animal Park, Hythe, Kent, Great Britain; Centre national de la recherche scientifique, FRANCE

## Abstract

Rehabilitation of animals followed by reintroduction into the wild can benefit conservation by supplementing depleted wild populations or reintroducing a species in an area where it has been extirpated or become extinct. The western lowland gorilla (WLG, *Gorilla g. gorilla*) is persistently poached; infants are often illegally traded and used as pets. Some are confiscated and rehabilitated, then kept in sanctuaries or reintroduced into the wild. Prior to reintroduction, the ability of the orphans to survive independently in their environment needs to be assessed. Here, we performed a multivariate analysis, including diet composition, activity-budget, and pattern of strata using of a group of five juvenile WLG in the process of rehabilitation and distinguished three sub-periods of ecological significance: the high furgivory period, the *Dialium* fruits consumption period, and the high folivory period. The consequences of these variations on their well-being (play behaviour) and the group cohesion (spatial proximity and social interactions) were examined. Like wild WLGs, diets shifted seasonally from frugivorous to folivorous, while the same staple foods were consumed and large amounts of *Dialium* fruits were seasonally gathered high in trees. When succulent fruit intake was the highest, thus providing high energy from sugar, juveniles spent less time feeding, more time playing and group cohesion was the highest. Conversely, the cohesion decreased with increasing folivory, individuals spent more time feeding and less time playing together. Nonetheless, the group cohesion also decreased after the death of one highly social, wild-born orphan. This may underscore the importance of skilled individuals in the cohesion and well-being of the entire group and, ultimately, to rehabilitation success. This study evaluates the rehabilitation success with regards to the methods used and highlights the need to consider a set of individual and environmental factors for enhancing rehabilitation while preserving the local biodiversity and individual well-being.

## Introduction

For the past decades, loss and fragmentation of habitats, zoonotic diseases, poaching, ecological disaster, live animal trade and other human-related activities has led to the rapid decline of hundreds of species [1]. In response, many rehabilitation programmes have been created aimed at taking care of wild sick, injured or pet animals [2]. The rehabilitation of animals is defined as the process by which captives are ‘treated for medical and physical disabilities until they regain health, are helped to acquire natural social and ecological skills, and are weaned from human contact and dependence, such that they can survive independently (or with greater independence) in the wild’ [3]. Animals in rehabilitation may either come from the wild, where they have been rescued by humans or come from captive environments like zoological parks. It concerns species of birds (e.g. Puerto Rican parrots (*Amazona vittata*) [4]; African penguins (*Spheniscus demersus*), Cape gannets (*Morus capensis*) [5]), mammals (e.g. cheetah (*Acinonyx jubatus*) [6], golden lion tamarins (*Leontopithecus rosalia rosalia*) [7]), and even reptiles (star tortoise (*Geochelone elegans*)[8]). Rehabilitated animals may either be kept in a sanctuary providing a closed and protected area that serves as a permanent or long-term refuge, or be released into the wild so that they can live naturally again. There released animals can either serve as conservation tools to supplement depleted wild populations [9,10], or they can be reintroduced into an area where it had been previously extirpated or become extinct as long as the reasons why that species was extinct in that given area has been removed or controlled [3,11].

Rehabilitation centres and sanctuaries provide opportunities to study interspecies disease transmission, which is of interest to both conservationist and human medicine [12,13]. Centres are also effective tools for communication, sensitization and education to biodiversity conservation, animal well-being and health safety rules at both international and local scales. Centres provide job opportunities for local communities often living in remote areas of high poverty, and can be used as a tourism, possibly providing new sources of revenue. Therefore, Centres are also an effective way to protect the surrounding areas [14].

Conversely, rehabilitation programmes, sanctuaries, as well as release and reintroduction programmes raise several problems, risks and critics. First, the released individuals may experience unprecedented stress events in areas where wild conspecifics or predators threaten their survival, in particular in primate species: e.g. gibbons (*Hylobates agilis albibarbis*) and golden lion tamarins (*Leontopithecus rosalia rosalia*) [15]. Second, the wild population may be affected in the released areas if the available resources cannot sustain the increase in population (e.g. marine mammals [16]). Third, the transmission of diseases between caretakers and captive animals, as well as the risk of transmitting human pathogens to a susceptible and naïve wild population through released animals needs to be addressed and controlled with adequate hygiene and security policies. Due to the close genetic make-up of humans, the risks are higher in the case of non-human primates (e.g. sanctuary chimpanzee (*Pan troglodytes*) [17]). For these reasons, post-release monitoring of animal-behavioural ecology and health is necessary to estimate how successful both rehabilitation and released individuals have been.

Among mammals, primates is the order the most threatened by extinction. Over the past 20 past years, the number of threatened species has increased by 21% (20 new threatened species) [1]. In the Central African region alone, which harbours one of the most highly diverse biomass of primates in the world, monkeys are alarmingly hunted or poached, including all three African great apes (i.e. bonobo (*Pan paniscus*), chimpanzee (*Pan troglodytes*) and all species of gorillas (*Gorilla sp*.)) [18].

Among them, the western lowland gorilla (WLG; *Gorilla gorilla gorilla*) is a critically endangered species. First, Ebola hemorrhagic fever has been responsible for a mortality rate close to 95% in some areas of the Republic of Congo and Gabon [19,20] and is believed to have led to approximately 5,000 deaths overall in this species [21]. Second, poaching, despite being theoretically punishable by law, is still heavily occurring within the whole distribution area of the species [22], especially since law enforcement is most often lacking, and sentences are only rarely given or enforced [23]. Although anti-poaching actions may help reduce the hunting pressure, many WLGs are still killed, and infants captured alive, sold and used as pets in villages or urban areas [24,25]. This is not only a local phenomenon, but the reality of today is that there is an illegal international trade of young apes with some countries (especially South-East Asia) [26].

Whenever adequate facilities are available, confiscated young WLG orphans may be integrated into rehabilitation programmes [27]. Following lengthy pre-release phases, two groups composed of both wild-born and captive-born orphans were released by the Projet Protection des Gorilles (PPG) of The Aspinall Foundation into the Batéké Plateau National Park in Gabon in 2001 and 2004, with high post-release survival, successful reproduction, and female transfer between groups [28,29]. The released groups were made up of wild-born orphaned WLGs confiscated in Gabon and of captive-born individuals from the Howletts and Port Lympne Wild Animal Parks who had been rejected by their mothers [28,30,31].

Even if our understanding of wild WLG behaviour still remains quite thin, some data on diet, activity budget and pattern of strata use are available [32]. WLG diet is highly diverse; WLGs are not only plant consumers, feeding on leaves and all other parts of various plants, but they are also highly frugivorous during periods of high fruit availability [33–35]. They also consume large amounts of understory monocotyledonous plants, which provide important staple foods throughout the year, especially during periods of fruit scarcity, and which are subsequently essential for gorilla survival [32,34–36]. Insects are also eaten by WLG, in particular ants and termites [37,38].

Although few studies analyzed the activity-budgets of immature individuals, Masi et al. [32] showed that it varies with seasons and individuals (age and sex): during periods of high frugivory, WLGs spend less time feeding, as fruits have a high energy value. All year long, they spend much of their time feeding, and less time in social activities [32]. On the other hand, females and immatures spend more time eating and less time resting than mature males [32]. Meanwhile, they allocate more energy than males to social interactions that include social play between siblings and maternal care [32]. In the wild, WLGs form stable and cohesive groups led by one silverback male [39]. The frequency of contact is low, grooming being rare or absent, except between mothers and infants [32]. In adults, hooting vocalization is the most important social behaviour [32].

Finally, WLG is a terrestrial species, which travels on the ground, rests either on the ground or at low strata according to individuals and seasons, and feeds either at ground level or in trees [40].

Here, we studied the behavioural ecology of juvenile WLGs during their rehabilitation process through a multivariate analyses of their diet, activity budget and pattern of strata use, and evaluated their well-being accordingly. We finally represented the social association/interaction patterns in social networks used as descriptive graphical representations of the sociality of the group [41] to illustrate the influence of change of diet composition on the group cohesion. Our data was compared with data from the wild to estimate both the behavioural and social skills of the juveniles and to assess their fitness for future release into the wild.

## Materials and Methods

### A- Ethics Statement

The reintroduction programme in the Plateaux Batéké National Park has been legally approved through a signed "cahier des charges" between the Ministry of Water & Forests and Reforestation of the Government of Gabon and The Aspinall Foundation in July 1998.

No permit was required for this specific study as it was entirely based on data collected non-invasively during standard pre-release monitoring procedures. Rehabilitation, food provisioning, medical care, and safety and hygiene standards for human caretakers complied with the Guidelines for Nonhuman Primate Re-introductions of the IUCN/SSC Re-introduction Specialist Group [42], the Best Practice Guidelines for the Re-introduction of Great Apes [3] and the Pan African Sanctuary Alliance (PASA) guidelines including the “PASA Primate Veterinary Healthcare Manual”.

Gorillas were fed one to five times a day with varying amounts, in function of their weight, age and the amount of natural vegetation consumed on the basis of the PASA Primate Veterinary Healthcare Manual and the Nutritional requirements of Non-Human Primates. This supplementary food also allowed with oral medical treatments when necessary.

Medical care, as part of the rehabilitation process, was performed by veterinarians to whom gorillas were well-habituated so that it minimized stress, suffering and the impact of procedures. It was performed non-invasively and without anaesthetic (for instance while caretaker grooming or playing with gorillas) as far as possible unless gorillas were badly injured or wounded. In such a case, the use of appropriate sedation (orally) followed by anaesthesia was implemented and physical restraint was avoided.

For more details, see *Mode of Rearing and Medical Procedures* below. PPG Gabon is a PASA member sanctuary.

### B- The reintroduction site

#### The Projet Protection des Gorilles

**Fig 1:**
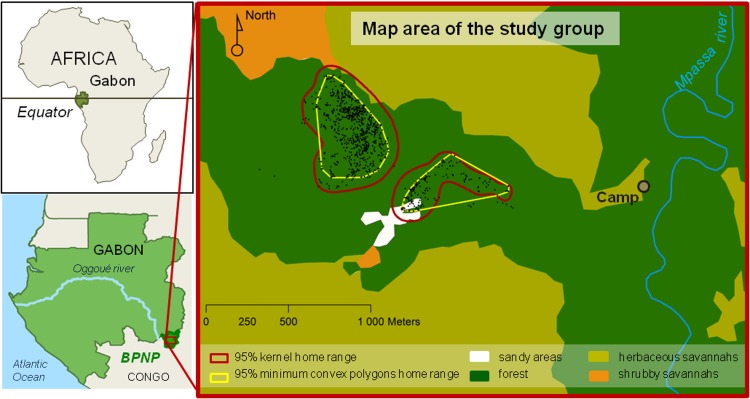
Location of the study site (left) and map area of the study group (right). Home range of the focal group for the entire study (February 2010 to October 2010) is presented in yellow (95% Minimum convex polygons) and in red (95% kernel home range) (using Garmin GPSMAP 60CSx and Biotas Version 2.0a, Ecological Software Solutions). The division of the study group home range in two areas resulted from the late use of the south-east area in September and October, after Bumbi death.

The gorilla reintroduction programme of the Projet Protection des Gorilles Gabon is located in the Batéké Plateaux National Park in the southeast of Gabon (S02.05, E14.05). The park consists of wooded and non-wooded grasslands intersected by the Mpassa River and its tributaries which are bordered by equatorial gallery and swamp forest (Fig. 1). Building on the experience of the WLG rehabilitation and reintroduction programme in the Plateaux Batéké region of the Republic of the Congo, in 1998 the Aspinall Foundation initiated a similar programme in Gabon [27,30]. No activity of wild WLGs was recorded in this area [25].

BPNP = Batéké Plateaux National Park.

#### Study period, study site and rehabilitation area

The study was undertaken from February to October 2010, and stretched through the minor dry season (January-March), the minor rainy season (April-June), the major dry season (July-September) and the major rainy season (October-December) [43–45]. The area used by the focal group during our study is free of wild or released WLGs and covers approximately 7.75ha with a mosaic of heterogeneous habitats including forest, forest edge, grassland and sandy areas (Fig. 1). A description of the major tree, shrub, herbaceous and liana species composing each habitat is shown in S1 Table.

### C- The gorillas

#### The group

The WLG group that was the focus of our study (Oudiki’s focal group) was composed of one male (Oudiki) and four females, with ages ranging from 1 to 4 years old at the beginning of the study. Among them, three individuals were hand-reared captive-borns transferred from the Howletts and Port Lympne Wild Animal Parks in the UK, and the two others were wild-born orphans confiscated in Gabon. Characteristics of each individual are presented in Table 1. The youngest individual (Bumbi) died before the end of the study, the 9th of September 2010, after being attacked by a wild chimpanzee (see Discussion).

**Table 1:** Characteristics of the members of the study group.

Individuals	Age during study (years)	Introduction date	Origin	Sex
Oudiki	4	Jun 2008	captive-born	M
Kouki	3	Jun 2008	captive-born	F
Lekoko	3	Mar 2009	wild-born	F
Tiya	2	Jun 2008	captive-born	F
Bumbi	1	Oct 2009	wild-born	F

In June 2008, Lekoko was first introduced to Oudiki, Kouki and Tiya, who was a few months old and hand-fed daily. Eventually, Bumbi joined the group in October 2009, four months before the beginning of this study.

#### Mode of Rearing and Medical Procedures

The rearing of the group was under close control of human caretakers. The group spent the night-time (from 4pm to 8am) protected from potential predators (leopards (*Panthera pardus*), chimpanzees (*Pan troglodytes*)) and from forest elephants (*Loxodonta africana cyclotis*) in a 4x4x3 m^3^ wooden cage, including platforms for nesting. The cage was enriched with fresh *Aframomum sp*. (Fam. Zingiberaceae) for feeding and nesting, and water daily. Depending on their age and ability to feed on forest vegetation, gorillas were either fed with milk products developed for human infants (Nestle, NAN), or cereal and milk based meals (Nestle, CERELAC). These meals were provided three times a day: twice a day in the cage before and after going into the forest respectively at 8am and 4pm, and once in the forest at noon. Gorillas spent the daytime in the natural environment (consisting mainly of forested area: Fig. 1) with at least two animal caretakers. Interactions with humans were not encouraged, but gorillas were not turned away when initiating contact. In this case, humans were instructed to behave like adult gorillas according to the situation and individuals involved (i.e. vocalizations and gestural). The training was directed by S.C., who has had great experience of wild-habituated gorillas and captive gorillas, and of wild-born orphans. Most of the time, the group displacements from patch to patch were initiated by caretakers, and the gorillas followed them. Both travel and resting times are thus highly under human influence. Individuals were not taught what food items to eat, and spontaneously consumed plants and insects. Health status of the gorillas was checked regularly (by H.DN) and they were dewormed every three months.

### D- Data collection

During daytime, behavioural data were all recorded by G.LF. following the instantaneous scan sampling method [46]. Data were collected every fifteen minutes on the five individuals during ten months from February to October 2010, corresponding to 484 hours of observation, and counting for 60.5 full-day monitoring (monthly mean = 6.72 ± 2.20 days) and 9755 scan samples. No focal sampling was done. Food items consumed during scan sampling were recorded and identified whenever possible. The ethogramm consisted of six activity categories: feeding, foraging, locomotion, social interactions between gorillas, social interactions with humans and resting. Among all six categories, we described a total of 127 different behavioural units, including 27 play behaviours (S2 Table). Solitary and energetic play behaviours were included in the locomotion category; play behaviours between gorillas were included in the category of social interactions between gorillas; play behaviours with humans in the category of social interactions with humans; and solitary and quiet play behaviours were included in the resting category. At any scan sample, distance of an individual to its nearest gorilla neighbour and to its nearest human was recorded in meters. To study the pattern of strata use, five classes of forest strata were considered: i) “ground level” at 0 metre, ii) “low stratum” beneath 2 m, iii) “intermediate stratum” from 3 to 5 m, iv) “high stratum” from 6 to 20 m and v) “canopy stratum” above 20 m.

### E- Statistical analysis

Statistical analyses were performed using R version 2.13.0 [47]. As slight differences in sample size among individuals and observation time existed over the nine months, data were weighed by the number of scans per hour per individual and per month (i.e. observation effort) prior to the analysis. Eventually, we considered every individual × month as a statistical individual (n = 43) displaying average monthly behaviours, keeping individual as conditioning variable in the analyses: each of the 43 data points was a single gorilla per month with the same gorillas being considered each month.

Firstly, the relationship between diet composition (DC; as response data set) and months (as explanatory factor or fixed variable) was investigated following the distance-based redundancy analysis (db-RDA), with gorilla individuals (n = 43) as a conditioning variable [48–50]. Eventually, the one-factor multivariate F# statistic [49] constructed by the RDA was tested by permutation (N. Perm = 9,999). Because only five individuals were studied, we did not aim at studying individual effects (i.e. male *vs* female, wild-born *vs* captive-born) for representative reasons. As the permutation test was significant (p<0.05), we investigated monthly diet composition (mDC; n = 9 months). Additionally, a cluster analysis was run to group similar months together, controlling for best grouping using the Calinski-Harabasz [51] criterion. The significance of the association of months within periods given by the cluster analysis was tested through an analysis of concordance, using Kendall’s coefficient together with a permutation test [50]. Secondly, following the same procedure described above for diet composition, a db-RDA was performed to examine the relationship between activity-budget (AB) and months, and the pattern of strata use (PSU) and months, with gorilla individuals as a conditioning variable. Also, cluster analysis was run for grouping months according to both AB and PSU. The db-RDA followed by the cluster analysis permitted to detect temporal variation in the three ecological data sets (DC, AB, PSU) and to group months within identical sub-periods. Thirdly, a Multiple CO-inertia Analysis (MCOA) [52,53] followed by Bonferroni’s *post hoc* test was performed on the basis of separate Principal Component Analysis (PCA) of monthly diet composition (mDC), monthly activity-budget (mAB) and monthly pattern of strata use (mPSU), to analyze simultaneously and to examine the relationships between these three ecological tables. It aims to identify the pattern of behavioural ecology according to the sub-periods found. The resulting "reference structure" defined the pattern of behavioural ecology by the study group. The efficiency of the MCOA was assessed through the squared covariance (cov2) between the scores of the three ecological tables and the scores of the reference, through the squared cosines of the angles (cos2) between the first two axis scores of the separate PCA and the first two axis of the reference, and through the RV coefficients [54]. RV coefficients measure the correlation between each of the three ecological tables and the reference structure, and were tested for significance using Mantel permutation tests [55]. Fourthly, a procrustean analysis was performed to examine the relationship between the pattern of behavioural ecology found by the MCOA and the structure of play behaviours. The matrix of play behaviours consisted of the four play variables (solitary and energetic play, play between gorillas, play with humans, and solitary and quiet play). The significance of the procrustean analysis was tested by a Mantel permutation test [56]. All tests were considered significant for pvalue<0.05 with Bonferroni’s adjustment when necessary.

#### R packages and functions used

Distance-based RDA procedures involved *vegdist*, *cmdscale*, *rda* and *anova*.*cca* functions available in 'vegan' package [57]. Cluster analyses involved *cascadeKM*, *kendall*.*global*, *kendall*.*post* functions of the package 'vegan' (K-means partitioning), *dist* and *hclust* functions of the package 'stats' (hierarchical clustering). The package 'stats' was used to perform Spearman’s product correlation tests using *cor*.*test* function and Wilcoxon tests were performed using *wilcox*.*test* function. The package 'ade4' [58,59] provided numerous functions for multivariate analysis: MCOA required *mcoa* function after creation of a K-table using the *ktab*.*list*.*dudi* function and the procrustean analysis needed the *procuste* function. Significances of RV coefficients and procrustean analysis were tested using *RV*.*rtest* and *procuste*.*randtest* functions.

### F- Social networks and group cohesion

Both social networks (i.e. graphs) of play relationships (interactions) and neighbours in close proximity (associations) were constructed on the basis of squared matrixes of dyadic relations [60], using the package 'sna' [61] of R version 2.13.0 [47]. Close proximities between one focal individual and its nearest neighbour were considered when the distance between them did not exceed two meters. Values of the matrixes were expressed as the proportion of time dyads were associated or interacted. The social networks were constructed for the three sub-periods of ecological significance: high frugivory period, *Dialium* fruits consumption period and high folivory period. Differences of mean interactions and associations between sub-periods were detected using an analysis of variance (ANOVA) followed by *post-hoc* Sidak adjustments, allowing for multiple pairwise comparisons between periods. Values are reported as means, and results were considered significantly different at pvalue<0.05. These analyses were performed using SPSS (version 17.0 for Windows; SPSS, Chicago, IL).

## Results

### A- Overview of the behavioural ecology

#### Characterization of diet, activity-budget and pattern of strata use

**Fig 2:**
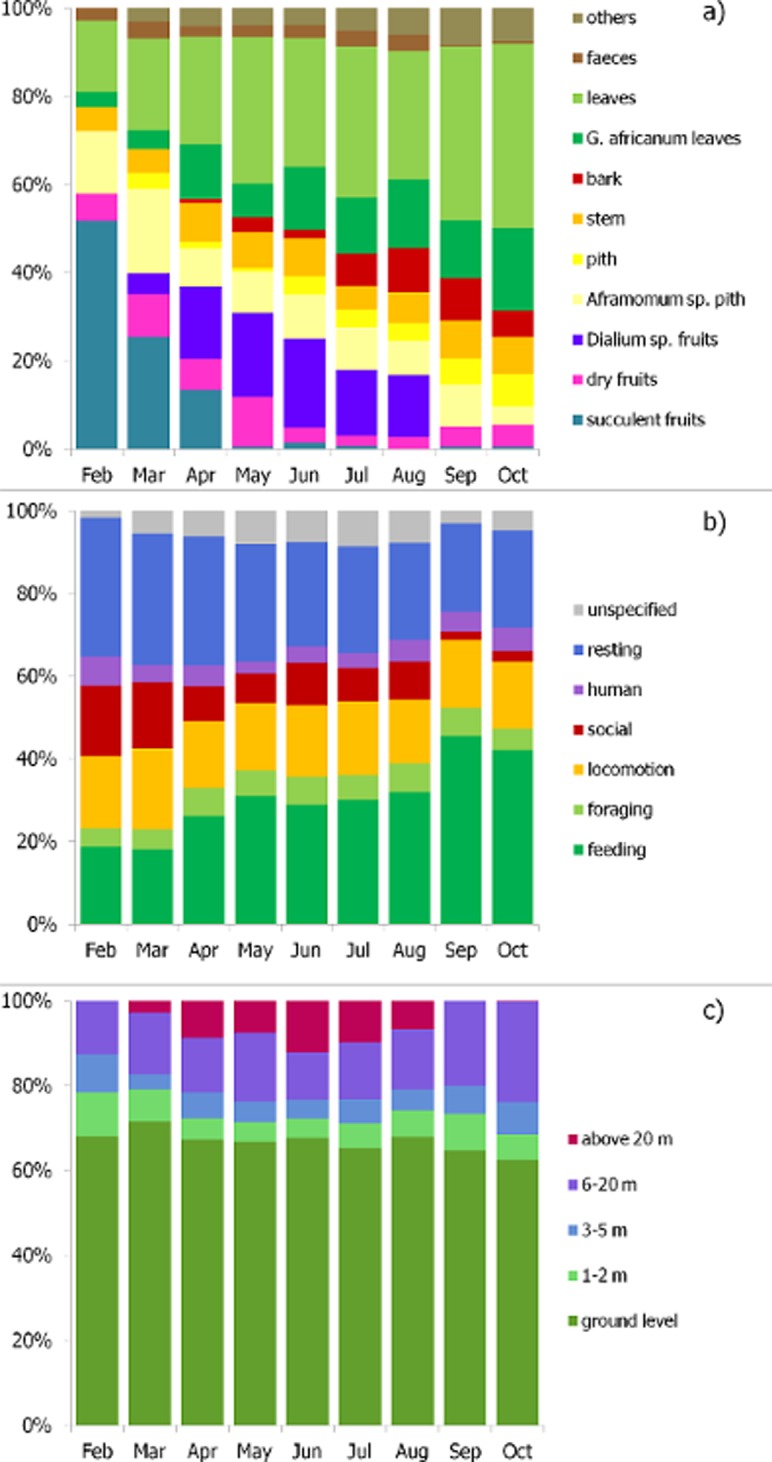
Monthly a) diet composition (mDC), b) activity-budget (mAB) and c) pattern of strata use (mPSU) of the study group in 2010. In Fig. 2b), unspecified behaviours are these that could not be described because of unclear visibility.

105 vegetal species were recorded to be consumed by the juvenile gorillas, among which 27 were successfully identified (S1 Table). The most frequently eaten species belonged to the families Apocynaceae, Arecaceae, Leguminosae-Caesalpinoideae, Commelinaceae, Euphorbiaceae, Gnetaceae, Marantaceae, Rubiaceae, and Zingiberaceae. Invertebrates, mostly ants, were sporadically consumed, mainly *Oecophylla longinoda* and *Crematogaster sp*. (mean = 1.2% ± 0.013 of the diet composition; max = 3.6% in September). An overall of eleven alimentary variables (i.e. items eaten) entered the matrix of diet composition, the monthly variation of which is presented in Fig. 2a. Three fruit variables were distinguished: succulent fruits such as of *Landolphia sp*. (Fam. Apocynaceae) or *Pachypodantium staudtii* (Fam. Annonaceae), dry fruits such as from the family Marantaceae, and, third, fruits of *Dialium sp*. alone (Fam. Leguminosae-Caesalpinoideae) because it is one of the main fruit trees of the area and represents the major part of frugivory in some months [32].

Activity-budget was as follows (Fig. 2b): feeding (18–45% of the time); foraging (4–7%); feeding and foraging times together (23–52%); locomotion (15–20%); social activities between gorillas (2–17%); interaction with humans (3–7%); resting (21–33%). Play behaviours between gorillas counted for 1.2–12.7% of the activity-budget and for 46.4–74.9% of social activities between gorillas. Pattern of strata use was as follows (Fig. 2c): “ground level” at 0 metre (62–71%); “low stratum” beneath 2 m (4–10%); “intermediate stratum” from 3 to 5 m (3–9%); “high stratum” from 6 to 20 m (11–24%); “canopy stratum” above 20 m and up to 40 m (0–12%).

#### Temporal variation

The db-RDA performed on the diet composition, activity-budget and pattern of strata use of every individual × month (i.e. statistical individual, n = 43) revealed a significant effect of months (n = 43, N. Perm = 9,999: F#_DC_ = 3.87, pvalue = 1e-04; F#_AB_ = 2.55, pvalue = 1e-04; F#_PSU_ = 1.53, pvalue = 0.00806, respectively). The cluster analysis gave the three identical and significantly different sub-periods for the three ecological variables: period 1, the high furgivory period, included February and March, period 2, the *Dialium* fruits consumption period, stretched from April to August and period 3, the high folivory period, comprised September and October (Kendall’s coefficients of diet composition grouping: W_DC1_ = 0.96, pvalue = 1.6e-04; W_DC2_ = 0.73, pvalue = 3e-05; W_DC3_ = 0.91, pvalue = 0.00162; activity-budget: W_AB1_ = 0.91, pvalue = 0.025; W_AB2_ = 0.95, pvalue = 3e-03; W_AB3_ = 0.98, pvalue = 6e-03; pattern of strata use: W_PSU1_ = 1, pvalue = 0.024; W_PSU2_ = 0.92, pvalue = 3e-03; W_PSU3_ = 0.95, pvalue = 0.034; n = 9, N. Perm = 9,999 for all). *A posteriori* permutation test confirmed that all months within periods were positively correlated (Spearman’s *rho* all positive, N. Perm = 999) [50].

#### Multitable analysis of behavioural ecological matrixes

**Fig 3:**
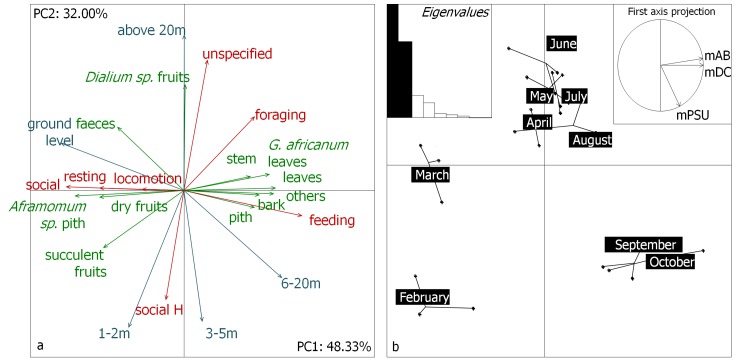
Multiple Co-inertia Analysis (MCOA): (3a) projection of the variables and (3b) projection of the months on the two first axis. In 5a, the variables of the diet composition table are in green, variables of the activity-budget table are in red and variables of the pattern of strata use table are in blue. The two first axis of the reference structure result from the simultaneous analysis of the three ecological tables and thus represents the habitat and resources use by the group. mDC = monthly diet composition; mAB = monthly activity-budget; mPSU = monthly pattern of strata use.

The first two axis of the reference structure of the MCOA, which determine the pattern of behavioural ecology of the study group, absorbed respectively 48.3% and 32.0% (sum = 80.3%) of the total covariance between the three ecological tables (mDC, mAB and mPSU) (Fig. 3). Square cosines (cos^2^), square covariance (cov^2^) and RV coefficients are presented in Table 2. Values of cos^2^ for the two first axes of the reference structure were high and homogeneous; hence the angles between the factorial axes of the PCA performed on the three ecological tables and the factorial axis of the reference structure were low. Subsequently, the ecological tables and the reference structure strongly matched. Because the sum of the values of cov^2^, as pseudo-eigenvalues, were similar for each table (ranging from 0.70 to 0.76), all tables participated equally to the construction of the reference structure. Also, RV coefficients were high, homogeneous and all statistically significant on the basis of the Mantel’s permutation tests (Table 2). On the first axis, mDC and mAB variables were well projected (cov^2^_mDC_ = 0.56; cov^2^_mAB_ = 0.50) and therefore highly correlated, while mPSU was better projected into the second axis (cov^2^_mPSU_ = 0.49) and thus very little correlated with each other. As well, the three time periods were well exhibited by the projection of the months, the first axis separating months mainly according to both DC and AB, and the second axis according to PSU (Fig. 3).

 

Unspecified behaviours are these that could not be described because of unclear visibility.

**Table 2:** The three statistics for the first two axes of the Multiple Co-inertia Analysis (MCOA).

Tables	Axis rank	Cos^2^	Cov^2^	RV coefficients	pvalues
mDC	1	0.98	0.56	0.81	1e-04
	2	0.94	0.14		
mAB	1	0.94	0.50	0.86	2e-04
	2	0.95	0.25		
mPSU	1	0.77	0.27	0.87	1e-04
	2	0.99	0.49		

RV coefficients tested by permutation (N.Perm = 9,999) were all significant (p<0.05), thus each of the three ecological data sets (see Fig. 3 for details) was highly correlated with the reference structure constructed by the MCOA.

In period 1 (February and March), the focal group’s diet incorporated a high amount of succulent fruits (38.6%), dry fruits (8.0%), *Aframomum sp*. pith (16.7%) while the proportion of total leaves was rather low (22.3%) and bark was not eaten. Meanwhile, gorillas strongly interacted with each other (16.5%), and feeding and foraging times together were low (less than a quarter of the activity-budget). The gorillas spent most of their time on the ground (69.7%), as they also did in periods 2 and 3 (respectively 67% and 63.6%), but, in period 1, they were rarely seen above 20 metres high (1.4%). The time allocated to play was the highest (27.3% of activities; for all significant Wilcoxon tests, see Table 3).

**Table 3:** Results of the comparisons between periods (Wilcoxon tests).

		Period 1-Period 2 (n = 35)	Period 1-Period 3 (n = 18)	Period 2-Period 3 (n = 33)
***Diet***				
	fruits of *Dialium sp*.	W = 3 (pvalue = 8.689e-06)	*ns*	225 (1.021e-05)
	succulent fruits	248 (4.985e-06)	80 (0.0003904)	*ns*
***Activity-budget***				
	feeding time	8 (7.299e-07)	10 (0.002988)	28 (0.000475)
	foraging time	23 (5.31e-05)	*ns*	*ns*
	social interactions	216 (0.0004621)	79 (0.004135)	183 (0.004738)
	unspecified behaviours	32 (0.0007305)	*ns*	193 (0.0009851)
***Pattern of strata use***				
	arboreal stratum (>20m)	5 (1.218e-05)	*ns*	225 (1.081e-05)
***Play***				
	play	227 (5.31e-05)	90 (2.165e-05)	185.5 (0.00465)
	social play	211 (0.001058)	84 (0.001638)	198.5 (0.0008412)

All not significant tests are noted "ns"; all others are significant.

Period 2, from April to August, was notably characterized by a larger amount of time feeding on fruits of *Dialium sp*. (16.9%) and an important use of the arboreal strata above twenty metres (9.0%). Feeding and foraging time increased from period 1, from 18.5% to 29.7% and from 4.6% to 6.5% respectively. Compared to period 1 (16.5%), the social interactions with conspecifics (8.6%) were reduced by half, and the total play in activities decreased to 11.8%. During this period 2, unspecified behaviours (i.e. behaviours that could not be described because of unclear visibility) were more frequent (7.7%) (Table 3).

During period 3, feeding time was the highest (43.8%). Foraging time in period 3 was not significantly different than in period 1 and period 2. Conversely the proportion of social interactions between gorillas was the lowest (2.3%). Social play counted for only 1.2% of the activities and was significantly lower than during the two previous periods (Table 3). Food consumed mainly consisted of leaves (53.2%), stem (8.0%), bark (7.4%), and only a few fruits (5.0%), especially succulent ones (0.3%).

In each period, stems and piths (including these of *Aframomum sp*.) varied insignificantly (5.5–8.5%, 12.0–18.5% respectively). Feces were eaten during periods 1 and 2.

In summary, period 1 was mainly characterized by the high amount of succulent fruits in the diet and by the high frequency of play behaviours and social interactions. Period 2 was primarily characterized by the use of the higher three stratum predominately for the consumption of *Dialium sp*. fruits. Period 3 was characterized by the highly foliviorous diet, by important feeding times and conversely by the low frequency of both play behaviours and social interactions.

Throughout our study, stretching from February to October 2010, the diet shifted from frugivorous to folivorous. In period 1 (February-March), which covered the minor dry season that is characterized by a high abundance of succulent fruits in the forest [44,62], succulent fruit intake was indeed higher than in periods 2 and 3 (Table 3). Fruit intake subsequently decreased continuously, conversely to the proportion of leaves. At the end of the major dry season (August, period 2), characterized by low availability of fruit species [43,62], fruit intake became dramatically low, while bark intake increased. By contrast, the proportion of stem and pith eaten by the focal group remained rather stable throughout the study. Interestingly, in period 2 (March-August), the consumption of *Dialium sp*. fruits replaced, at least partially, the succulent fruit intake that progressively decreased. Accordingly, the pattern of strata use changed, and the group spent more time above 20 meters (up to 40 meters), because *Dialium sp*. fruit-producing trees are tall. Although at this high stratum the visibility was unclear due to the dense undergrowth, therefore resulting in behaviours being unspecified about half the time, the behavioural data successfully collected showed higher proportion of feeding, especially on *Dialium* fruits (data available on request), similarly to wild counterparts [40].

### B- Relation between behavioural ecology and well-being

#### Procrustean analysis: linking behavioural ecology to play behaviours

**Fig 4:**
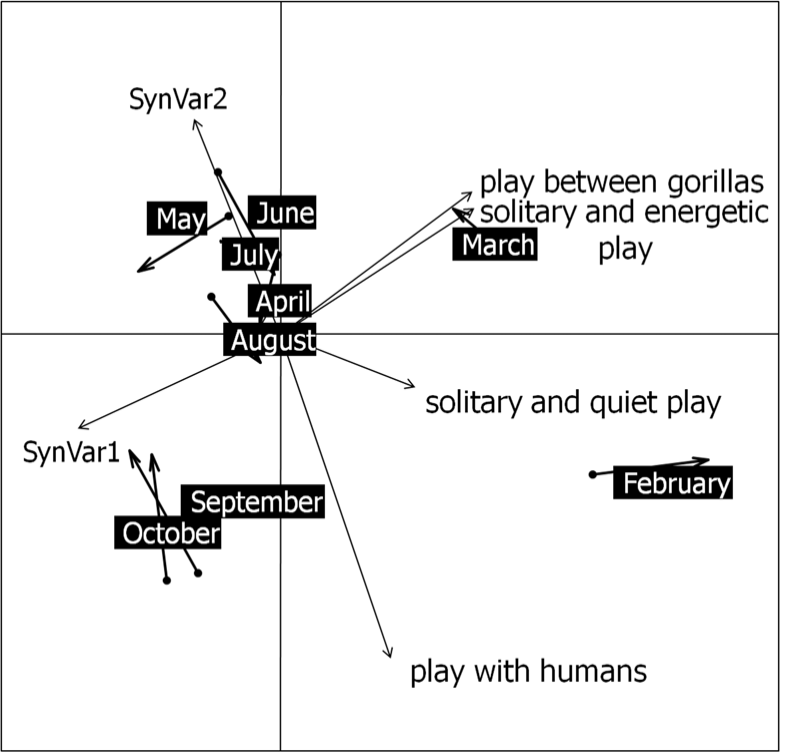
Procustean analysis between the two first axes (SynVar1; SynVar2) of the reference structure (i.e. compromise) obtained from the multiple co-inertia analysis of the three behavioural ecological tables and the play behaviours.

Play behaviours accounted for a large part of the entire between-gorillas social interactions (60.1 ± 9.4%). The permutation test showed that the procrustean analysis was significant (observed value = 0.70, N. replicates = 9,999, pvalue = 0.0015), thus that the relation was statistically strong between play behaviours (Fig. 4) and the pattern of behavioural ecology synthesized by the MCOA. The graphical representation of the procrustean analysis revealed that all play behaviours (i.e. solitary and energetic play; solitary and quiet play; play between gorillas; and play with humans) occurred more often in the period of high frugivory (period 1: February and March) (Table 3), the only period including months with positive values on the first axis of the projection (Fig. 4). Proportions of total play and between-gorillas play both decreased through months and were positively correlated to the proportion of social interactions but negatively to feeding proportion, negatively correlated to the proportion of leaves eaten including *G*. *africanum* leaves and other leaves eaten. On the contrary, they were positively correlated with the proportion of succulent fruits eaten (All significant Spearman's *rho* tests are presented in table 4).

February and March are the only two months with positive values on the first axis and the two with the highest proportion of play behaviours in activity-budget.

**Table 4:** Results of the Spearman’s product correlation tests between play variables and ecological variables.

*Play variables*	*Ecological variables*	*rho*	*S (pvalue)*
total play	social interactions	+0,85	S = 2036 (pvalue = 4e-13)	
social play	social interactions	+0,9	1270.548 (1e-16)	
total play	feeding	-0,79	23719.4 (3e-10)	
social play	feeding	-0,77	23503.6 (1e-09)	
total play	*G*. *africanum* leaves	-0,37	18104.02 (0.0155)	
social play	*G*. *africanum* leaves	-0,46	19335.2 (0.0019)	
total play	other leaves	-0,64	21707.3 (4e-06)	
social play	other leaves	-0,63	21523.3 (7e-06)	
total play	succulent fruits	+0,61	5131.8 (1e-05)	
social play	succulent fruits	+0,51	6530.9 (0.0005)	

All tests are significant.

#### Group cohesion illustration: social network of play interactions and proximity association

**Fig 5:**
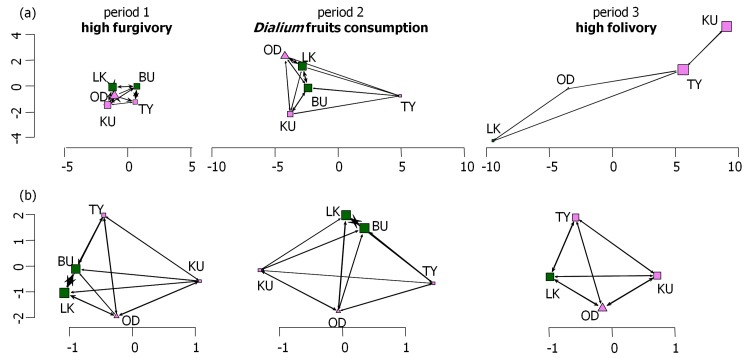
Social networks of (a) play interactions between gorillas and of (b) close proximity between individuals and their nearest neighbour, for each of the three behavioural ecological periods. The social networks are scaled for comparisons between periods. Scales of (a) and (b) are not comparable. Period 1 of high frugivory and play comprises February and March, period 2 of Dialium fruits consumption stretches from April to August and period 3 of high folivory comprises September and October 2010. BU: Bumbi (dead in period 3); KU: Kouki; LK: Lekoko; OD: Oudiki; TY: Tiya Square vertices: females (BU, KU, LK, TY); triangular vertices: male (OD) Pink vertices: captive-born gorillas (KU, OD, TY); green vertices: wild-born gorillas (BU, LK)

Through the study, the frequency of play interactions among the group decreased. The ANOVA performed on play interactions showed that there was a significant difference between periods (F = 7.279, df = 69, pvalue = 0.001). The Sidak test then showed that the mean difference during the period of high frugivory (period 1) was significantly higher, therefore the group was more socially cohesive than during the period of high folivory (period 3), when play interactions occurred sporadically after Bumbi died. As a result, the social networks of play interactions enlarged (i.e. distance between individuals increased) from period 1 to period 3, that is individuals interacted less strongly with each other (Fig. 5a). In period of *Dialium* fruits consumption (period 2), the frequency of play interactions decreased but not significantly. Regarding the social networks of close proximity with the nearest neighbour (Fig. 5b), the ANOVA detected no significant differences between periods, that is the social network of spatial proximity did not enlarge significantly between periods and the spatial cohesion remained stable. However, at the individual scale, it appears that Bumbi and Lekoko (wild-born orphans) constituted the most associated dyad during period 1 of high frugivory and period 2 of *Dialium* fruits consumption, being subsequently the most central individuals (period 1: eigenvector centrality scores of network position [63] evcent = 0.61 and 0.64, respectively for Bumbi and Lekoko; period 2: evcent = 0.66 and 0.64, respectively). Additionally, both also appear frequently as the nearest neighbour of the three other captive-born orphans. After Bumbi’s death on the 9th of September at the beginning of the period 3, all values of evcent were nearly equal, and preferred associations became less obvious regarding both distance between dyads and thickness of their link.

The thickness of a link (edge) represents the strength of the association/interaction between two individuals (vertices), while the size of the arrows gives the intensity of the association/interaction from one to another. The eigenvector centrality scores of network positions [63] (evcent) were calculated and only given as descriptive statistics, more central individuals (i.e. with higher scores) being represented by larger vertices.

## Discussion

Diet composition, activity-budget and pattern of strata use were the three ecological tables investigated to distinguish three ecologically homogeneous periods: high frugivory period, *Dialium* fruits consumption period and high folivory period. The consequences of these variations on the play behaviour (used as proxy of well-being) and the sociality (cohesion of the group and relationships between individuals) of the orphans in rehabilitation were examined.

The shift from a highly frugivorous to a folivorous diet during periods of fruit scarcity is in accordance with previous reports on wild WLGs [32,64–68]. First, wild WLGs also increase their bark consumption, especially of the family Apocynaceae [35,37,65], in the same period of fruit scarcity; second, they also consume large amounts of terrestrial herbaceous vegetation all year long, such as Commelinaceae, Marantaceae or Zingiberaceae piths that represent their staples foods [35,36,69]; third, *Dialium sp*. fruits are extensively consumed by wild WLGs in years of high availability, representing the major part of frugivory in some months [32]. Interestingly, the group also consumed the same ant species as wild gorillas [70]. Therefore, the diet composition and variation of the group was similar to that of wild WLGs whereas they were not taught by humans what to feed on. Individuals may have benefited from the experience of each other, especially from wild-borns, but they may also have learnt spontaneously by tasting. The part of trial and error (innovation) and social transmission of knowledge, though of interest [71], has not been evaluated here.

Furthermore, the more the group fed on succulent fruits, the less they allocated time for feeding, and the more time they spent in social interactions, essentially consisting of play. In wild juvenile primates, consumption of large amounts of succulent fruits is also associated with short feeding time and, conversely, longer (social) play time, probably because high-energy fruits tend to increase excitement and stimulate all forms of social behaviour, especially play (gelada baboons (*Theropithecus gelada*): [72]; WLGs: [32]). Additionally, the procrustean analysis showed that high fruit intake was not only associated with high levels of social play but with all forms of play behaviour. This finding reinforces the previous assumption that sugary fruits stimulate young gorillas to play together or alone, strengthening their social link and their well-being [73–75]. The social networks pictured this observation, since, in period of high frugivory and play (period 1), individuals were highly associated (close proximity network analysis) and often interacted together (social play network). On the contrary, during the period of *Dialium* fruits consumption (period 2) and even more during the folivory period (period 3), the frequency of play behaviours dramatically decreased and the structure of the group became less cohesive (lower spatial proximity, less interactions). This finding of lower cohesion among group members during the low fruit season is contrary to wild WLG findings [32]. In the wild however, the group cohesion is lower during frugivory period because the group spread to gather more dispersed food resources [34].

Because of human influence on gorillas displacements from patch to patch, both resting and travel times, which remain rather stable, are not discussed. In the wild, high frugivory would be associated with an increase in travel time in WLG [32,68].

Overall, the group time spent feeding and foraging was lower than for wild immature (23–52% compared to 61.1–76.1% in the wild: [32]). This difference may be explained by the fact that the study group were fed three times a day with milk, so that their energetic requirements from natural foods may also be lower. Resting (21–33%) and travel times (15–20%) were twice the times observed in wild immature counterparts (respectively 11.1–12.3% and 9.0–11.4%: [32]), but highly under human influence. On the other hand, time invested in social activities (2–17%) was quite similar to wild immatures (maximum in the wild of 16.5%: [32]). However wild immatures spend nearly all of their social time playing (90–95%: [32]), whilst during our study it never represented more than 75% of social interactions; although total social playing time (1.2–12.7%) was similar or even higher than for wild counterparts (1–4% in the wild: [32]). The absence of adults in the group may have stimulated the older immatures to behave more like adults, which resulted in less playful interactions. For example, Lekoko used to protect Bumbi from Oudiki’s playing, or invited her to grab hold of her back or hips and to walk in contact with her. This point, however, needs further investigation.

The pattern of strata use of our study group underlined their terrestriality. As humans stay on the ground, this result has to be taken with caution. In the wild, WLGs change their activity patterns in response to changes in the diet [32]. Similarly, in our case, when *Dialium sp*. fruits where available, the gorillas used the highest stratum often to feed on them.

Ecologically speaking, the rehabilitation process appears to be successful, the orphans using their environment and interacting socially in a similar way to wild gorillas, but the influence of humans in their activity-budget remains high. This influence is however necessary to have better control on young individuals, to provide better protection to them and to slowly stimulate them to become less dependent on humans.

The period 3 (September-October: period of folivory) was particular in many ways and the interpretation of our observations raises some issues. One individual (Bumbi) died after being attacked by a wild chimpanzee. After this incident, individuals were poorly associated and rarely interacted, causing a reduction in the group cohesion. It is not possible to detect the respective role of each phenomenon but the shift toward a folivorous diet together with Bumbi's disappearance appeared to have directly affected the group dynamics and activity pattern.

All individuals witnessed the aggression of the adult male chimpanzee, which happened in tree and lasted for a few hours until Bumbi let herself fall from a branch to escape. The aggression consisted of violent smashing on trunk, then on biting and hitting, which was a highly stressful event for all others. After Bumbi stopped defending herself and vocalizing, the chimpanzee rested near her, sometimes hitting or biting her again. Her fingers and ears were deeply cut, her abdomen was opened. During the aggression, the rest of the group was grouped around the caretakers, who tried to intervene and intimidate the chimpanzee, however they were not successful. To reduce the stress, two caretakers moved with the gorillas to another area, while two others tried to get Bumbi back. This might be the only case on record of a chimpanzee attacking and killing a gorilla in a wild context. However, it stresses the need to precisely estimate the local chimpanzee population size and the subsequent risk to introduce gorilla orphans into chimpanzee territories, peripheral or core.

But the reasons why Bumbi's disappearance has reduced the group cohesion might be explained in a number of different manners. First, subsequently to Bumbi’s death, the area with the highest density of wild chimpanzees was avoided, and another area further east was used instead (Fig. 1). There, the gorillas may have found the need to discover and accommodate a new environment stressful. Second, the disappearance of an individual (Bumbi) decreased the different possibilities of interactions and associations. Third, Bumbi’s young age could have made her the centre of the attention of the others. Fourth, as playing frequency varied a lot in wild gorillas according to age, older infants decrease their feeding while when becoming juveniles they increase it again, possibly explaining the observed variation. Fifth, being wild-born could have made her more socially skilled to maintain the group cohesion. Indeed, in period 2 (period of *Dialium* fruits consumption), Bumbi became the most central individual, followed by Lekoko in both social networks, these two wild-borns formed the preferential dyad of social play interactions and were obviously highly central in the social network (Fig. 5).

Though not investigated here, individual variations in experience may be of crucial importance in such rehabilitation projects and identifying variations between individuals according to their age, sex and life history is important.

The time wild-borns lived with their wild counterparts before being captured or the time spent in captivity before rehabilitation may affect gorillas’ skills and abilities to survive [28]. In our case, it is likely that wild-born orphans were more experienced than captive-borns and therefore are more aware of what food to gather and that this knowledge was transmitted to others. Skilled individuals may also play an important role in maintaining the cohesion and well-being of the entire group.

Given their implications in ecology and evolution [76–78], individual variations in social competence, cognitive traits (e.g. problem-solving, risk sensitivity, choosiness) and personality traits (e.g. boldness, aggressiveness, exploration tendency) is one major point to shed light on orphan rehabilitation as experienced wild-born individuals can enhance the skills of the others. However, since many wild infant gorillas are captured each year [23], since they appear more suitable to be released into the wild and less susceptible to stress than captive-borns, and since human and financial resources are limited, top priority should be the rehabilitation of illegally captured wild-born infant gorillas and, in general, wild-born rescued animals.

As underlined by Sarrazin and Barbault [79], though “reintroduction success [is] linked to biopolitical conditions and long-term funding (…), once funding and local agreement are obtained, the success of reintroduction remains a question of population viability involving demographic, genetic, behavioural and ecological processes”, which therefore needs to be further studied [79].

## Supporting Information

S1 Table Non exhaustive list of the main vegetal species present within the three habitats composing the study area and parts eaten. (PDF)

S2 Table Ethogramm of the 127 different behavioural units considered within every 6 activity categories. (PDF)
