# Supplementary material for: Behavioural Ecology and Group Cohesion of Juvenile Western Lowland Gorillas (Gorilla g. gorilla) during Rehabilitation in the Batéké Plateaux National Park, Gabon
Source: PLoS One. 2015 Mar 26;10(3):e0119609. doi: 10.1371/journal.pone.0119609 (PMC4374851; doi:10.1371/journal.pone.0119609)
Supplement: S1 Table — (PDF) [file pone.0119609.s001.pdf]

| Types of Plants | Families | Species | Habitats | | |
|---|---|---|---|---|---|
| | | | FOR | EDG | SVN |
| **Trees** | Agavaceae | *Dracaena sp.* (F) | X | | |
| | Annonaceae | *Pachypodantium staudtii* (F) | X | | |
| | | *Xylopia aethiopica* | X | X | |
| | | *Xylopia hypolampra* | X | X | |
| | | *Xylopia staudtii* (F) | X | | |
| | Burseraceae | *Aucumea klaineana* (Ls) | X | X | |
| | | *Dacryodes sp* (F,Ls) | X | | |
| | | *Santiria trimera* (F,Ls) | X | | |
| | Caesalpinoideae | *Dialium sp.* (F,L) | X | | |
| | Mimosoideae | *Pentaclethra eetveldeana* | X | X | |
| | Olacaceae | *Ongokea gore* (F) | X | | |
| | Sapotaceae | *Omphalocarpum procerum* | X | | |
| **Shrubs and small trees** | Annonaceae | *Annona senegalensis* | | | X |
| | Euphorbiaceae | *Hymenocardia acida* (Fl,L,S) | | X | X |
| | | *Hymenocardia ulmoides* (L) | X | X | |
| | Flacourtiaceae | *Caloncoba welwitschii* (F) | | X | |
| | Passifloraceae | *Barteria fistulosa* | X | X | |
| | Rubiaceae | *Psychotria sp.* (F) | X | | |
| **Ligneous lianas** | Apocynaceae | *Landolphia sp.* (B,F,Ls) | X | X | |
| | Arecaceae | *Ancistrophyllum secundiflorum* (F,P) | X | X | |
| | | *Eremospatha haullevilleana* (B,L,P) | X | X | |
| | Euphorbiaceae | *Manniophyton fulvum* (L,P) | X | X | |
| | Gnetaceae | *Gnetum africanum* (L) | X | | |
| **Herbaceous lianas** | Commelinaceae | *Palisota schweinfurtii* (F,L) | X | | |
| | Marantaceae | *Hypselodelphis violacea* (F,P) | X | | |
| | | *Trachyphrynium braunianum* (F,P) | X | | |
| **Herbaceous plants** | Commelinaceae | *Palisota hirsuta* (F) | X | X | |
| | | *Palisota mannii* (F,L) | X | | |
| | Marantaceae | *Megaphrynium macrostachyum* (F,L,P) | X | X | |
| | | *Halopegia azurea* (F,L,P) | X | X | |
| | Melastomataceae | *Heterotis decumbens* (F) | | X | |
| | Zingiberaceae | *Aframomum sp.* (F,Fl,P,R) | X | X | |
| | | *Costus sp.* (Fl,L,St) | X | X | |

**Supplementary table 1. Non exhaustive list of the main vegetal species present within the three habitats composing the study area and parts eaten.**

Legend: Habitat: FOR = gallery forest; EDG = edge of forest; SVN = savannah

Plants parts eaten: B=bark; F=fruit; Fl=flower; L=leaf; Ls=leaf sheath; P=pith; R=roots; S=seeds; St=stem
