# Supplementary material for: Behavioural Ecology and Group Cohesion of Juvenile Western Lowland Gorillas (Gorilla g. gorilla) during Rehabilitation in the Batéké Plateaux National Park, Gabon
Source: PLoS One. 2015 Mar 26;10(3):e0119609. doi: 10.1371/journal.pone.0119609 (PMC4374851; doi:10.1371/journal.pone.0119609)
Supplement: S2 Table — (PDF) [file pone.0119609.s002.pdf]

#	Behavioural units	Social context	Additionnal description	Corresponding activities
001	drinking	none	self-explanatory	Feeding
002	ingesting	none	self-explanatory	Feeding
003	licking	none	self-explanatory	Feeding
004	pulling a branch or a stem towards on	none	self-explanatory	Foraging
005	scratching a termitarium	none	self-explanatory	Foraging
006	digging soil	none	self-explanatory	Foraging
007	harvesting	none	self-explanatory	Foraging
008	investigating a dead branch or trunk	none	self-explanatory	Foraging
009	moving a branch, a stone, a leave…	none	self-explanatory	Foraging
010	opening a fruit	none	self-explanatory	Foraging
011	peeling a fruit	none	self-explanatory	Foraging
012	scratching soil, trunk or fruit skin	none	self-explanatory	Foraging
013	smelling a food item	none	self-explanatory	Foraging
014	smelling soil	none	self-explanatory	Foraging
015	tearing a leave up	none	self-explanatory	Foraging
016	thining leaves out	none	self-explanatory	Foraging
017	uprooting or pulling a branch up	none	self-explanatory	Foraging
018	brachiation	none	self-explanatory	Locomotion
019	climb a tree or liana	none	self-explanatory	Locomotion
020	climb down	none	self-explanatory	Locomotion
021	clung to a trunk	none	self-explanatory	Locomotion
022	hanging from a branch or liana	none	self-explanatory	Locomotion
023	nesting	none	self-explanatory	Locomotion
024	pounding the ground or a trunk for playing	none	self-explanatory	Locomotion
025	run	none	self-explanatory	Locomotion
026	self-playing	none	self-explanatory	Locomotion
027	self-playing and rolling	none	self-explanatory	Locomotion
028	self-playing and turning round on itself	none	self-explanatory	Locomotion
029	self-playing eyes closed	none	self-explanatory	Locomotion
030	self-playing hung from a branch or liana	none	self-explanatory	Locomotion
031	slinding down	none	self-explanatory	Locomotion
032	smashing/dragging plants for playing	none	self-explanatory	Locomotion
033	swinging from a branch or liana	none	self-explanatory	Locomotion
034	walking	none	self-explanatory	Locomotion
035	playing with a tool (stick, leave, sand…)	none	Locomotion or Resting according to the energy used in the play	Locomotion/Resting
036	quadrupedal standing for monitoring	none	self-explanatory	Resting
037	bipedal standing	none	self-explanatory	Resting
038	bipedal standing for monitoring	none	self-explanatory	Resting
039	lying	none	self-explanatory	Resting
040	lying near another	none	self-explanatory	Resting
041	monitoring while lying	none	self-explanatory	Resting
042	monitoring while seated	none	self-explanatory	Resting
043	playing gently in bipedal standing	none	self-explanatory	Resting
044	playing gently while in quadrupedal standing	none	self-explanatory	Resting
045	playing gently while lying	none	self-explanatory	Resting
046	playing gently while seated	none	self-explanatory	Resting
047	quadruedal standing near another	none	self-explanatory	Resting
048	quadrupedal standing	none	self-explanatory	Resting
049	resting near another	none	self-explanatory	Resting
050	resting seated	none	self-explanatory	Resting
051	seated on a nest	none	self-explanatory	Resting
052	self-grooming	none	self-explanatory	Resting
053	sleeping lying	none	self-explanatory	Resting
054	sleeping seated	none	self-explanatory	Resting
055	climbing a tree towards another	Affiliative behaviour	self-explanatory	Social with gorillas
056	climbing down a tree to another	Affiliative behaviour	self-explanatory	Social with gorillas
057	clutching at another	Affiliative behaviour	self-explanatory	Social with gorillas
058	flowing in walking	Affiliative behaviour	self-explanatory	Social with gorillas
059	following another in brachiation	Affiliative behaviour	self-explanatory	Social with gorillas
060	following another in climbing down	Affiliative behaviour	self-explanatory	Social with gorillas
061	following another in climbing up	Affiliative behaviour	self-explanatory	Social with gorillas
062	following another in running	Affiliative behaviour	self-explanatory	Social with gorillas
063	grooming	Affiliative behaviour	self-explanatory	Social with gorillas
064	hanging on another	Affiliative behaviour	self-explanatory	Social with gorillas
065	holding on another	Affiliative behaviour	self-explanatory	Social with gorillas
066	intervening with	Affiliative behaviour	one individual supports one of the two individuals engaged in an agonistic interaction.	Social with gorillas
067	resting in contact	Affiliative behaviour	two individuals resting together in physical contact, exclusive of other behaviours	Social with gorillas
068	resting in ventro-dorsal contact	Affiliative behaviour	self-explanatory	Social with gorillas
069	resting in ventro-ventral contact	Affiliative behaviour	self-explanatory	Social with gorillas
070	rolling towards another	Affiliative behaviour	self-explanatory	Social with gorillas
071	running to another for protection	Affiliative behaviour	self-explanatory	Social with gorillas
072	touching another	Affiliative behaviour	self-explanatory	Social with gorillas
073	walk towards another	Affiliative behaviour	self-explanatory	Social with gorillas
074	walking clutched at another hips	Affiliative behaviour	self-explanatory	Social with gorillas
075	walking with another clutched at its hips	Affiliative behaviour	self-explanatory	Social with gorillas
076	attempt at stealing (including food)	Agonistic behaviour	self-explanatory	Social with gorillas
077	food-stealing	Agonistic behaviour	self-explanatory	Social with gorillas
078	agression with contact	Agression with physical contact	includes a combination of biting, dragging, hiting, shoving	Social with gorillas
079	biting	Agression with physical contact	self-explanatory	Social with gorillas
080	dragging another	Agression with physical contact	self-explanatory	Social with gorillas
081	hiting another	Agression with physical contact	self-explanatory	Social with gorillas
082	shoving	Agression with physical contact	self-explanatory	Social with gorillas
083	agression without contact	Agression without physical contact	includes a combination of chasing, lunging, open-mouthed threat face…	Social with gorillas
084	chasing	Agression without physical contact	self-explanatory	Social with gorillas
085	lunging	Agression without physical contact	self-explanatory	Social with gorillas
086	open-mouthed threat face and threatening movements towards another	Agression without physical contact	self-explanatory	Social with gorillas
087	pig-grunting vocalization	Agression without physical contact	self-explanatory	Social with gorillas
088	running at another	Agression without physical contact	self-explanatory	Social with gorillas
089	chest-beating directional or non-directional	Communication - Display	to be distinguished from play behavior by the aggressive context and lack of play face	Social with gorillas
090	displaying	Communication - Display	an excited performance including chest beating, hooting, smashing/dragging plants, pounding the ground, symbolic feeding	Social with gorillas
091	hooting	Communication - Display	self-explanatory	Social with gorillas
092	pounding the ground	Communication - Display	self-explanatory	Social with gorillas
093	smashing/dragging plants	Communication - Display	self-explanatory	Social with gorillas
094	strut stance	Communication - Display	self-explanatory	Social with gorillas
095	symbolic feeding	Communication - Display	self-explanatory	Social with gorillas
096	anticipating food vocalization	Communication - Vocalization	a vibration vocalization performed by individuals immediately before meals, distinguished from belch vocalization by the context.	Social with gorillas
097	appeasement vocalization	Communication - Vocalization	a whiny vocalization given in response to agonistic behaviour	Social with gorillas
098	belch vocalization	Communication - Vocalization	an expression of contentment	Social with gorillas
099	moaning against another	Communication - Vocalization	a vocalization performed by one individual that another is about to interact with. This vocalization was especially perfomed by one individual (Tiya) against the male Oudiki that intended to play with her, but often roughly	Social with gorillas
100	scream vocalization	Communication - Vocalization	a high-pitched, high volume scream most often used in tense or aggressive situations	Social with gorillas
101	stress hooting	Communication - Vocalization	hooting performed in a stress situation	Social with gorillas
102	mounting	Dominance behaviour	one individual climbs on top of another individual, dorsally or ventrally, distinguished from play	Social with gorillas
103	agressive play	Play behaviour	during social playing, one or more individuals start escalading agressiveness. It could result in agression.	Social with gorillas
104	invite to play with contact (dragging, pushing…)	Play behaviour	self-explanatory	Social with gorillas
105	invite to play without contact (play displaying, initiating play chasing, …)	Play behaviour	self-explanatory	Social with gorillas
106	mock-bitting with another	Play behaviour	self-explanatory	Social with gorillas
107	play boxing	Play behaviour	self-explanatory	Social with gorillas
108	play chasing	Play behaviour	self-explanatory	Social with gorillas
109	play chest-beating	Play behaviour	self-explanatory	Social with gorillas
110	play wrestling	Play behaviour	self-explanatory	Social with gorillas
111	playing and laughing with another	Play behaviour	self-explanatory	Social with gorillas
112	playing while hanging on a branch or liana	Play behaviour	self-explanatory	Social with gorillas
113	playing with another	Play behaviour	self-explanatory	Social with gorillas
114	pretending to be blind	Play behaviour	one individual close his eyes and moves blind to another. This often follows an exciting play.	Social with gorillas
115	avoiding another	Submissive behaviour	self-explanatory	Social with gorillas
116	fleing another	Submissivebehaviour	self-explanatory	Social with gorillas
117	cowering down	Submissive behaviour	one individual adopt this submission posture to be protected from agression or rough play	Social with gorillas
118	smelling another	Undetermined type	self-explanatory	Social with gorillas
119	climbing on human to be transported	Affiliative behaviour	self-explanatory	Social with humans
120	following human	Affiliative behaviour	self-explanatory	Social with humans
121	hanging on human	Affiliative behaviour	self-explanatory	Social with humans
122	transported by human	Affiliative behaviour	self-explanatory	Social with humans
123	mock-biting	Play behaviour	self-explanatory	Social with humans
124	playing and laughing	Play behaviour	self-explanatory	Social with humans
125	playing with human	Play behaviour	self-explanatory	Social with humans
126	playing with human eyes closed	Play behaviour	self-explanatory	Social with humans
127	playing with human hung from a branch	Play behaviour	self-explanatory	Social with humans

Supplementary table 2. Ethogramm of the 127 different behavioural units considered within every 6 activity categories.
